# Use of electronic devices in leisure time modifies the prevalence and factors associated with sedentary behavior derived exclusively from excessive television viewing among Brazilian adults

**DOI:** 10.1186/s12889-023-16517-7

**Published:** 2023-08-23

**Authors:** Cecília Bertuol, Murilo Henrique Corrêa da Silveira, Rodrigo de Rosso Krug, Juliedy Waldow Kupske, Grégore Iven Mielke, Giovani Firpo Del Duca

**Affiliations:** 1 Graduate Program in Physical Education, Federal University of Santa Catarina, Campus Universitário Reitor João David Ferreira Lima, Florianópolis, SC 88040-900 Brazil; 2 Graduate Program in Integrative Health Care, University of Cruz Alta, Cruz Alta, RS 98020-290 Brazil; 3 Graduate Program in Human Movement Science, Federal University of Rio Grande do Sul, Rua Felizardo 750, Porto Alegre, RS 90690-200 Brazil; 4 School of Public Health, The University of Queensland, Brisbane, QLD 4006 Australia

**Keywords:** Leisure activities, Television, Computers, Hand-held devices, Cell phones, Cross-sectional studies

## Abstract

The growing interest in the study of sedentary behavior is justified by its increasing presence in people's daily lives, particularly in leisure time. The aim of this study was to compare the prevalence and factors associated with sedentary behavior derived exclusively from TV time and from its combination with the time spent using other electronic devices among Brazilian adults (*n* = 52,443). This cross-sectional study used data from the Vigitel survey (2019), which included subjects ≥ 18 years old who resided in the capitals of the 26 Brazilian states and Federal District. High TV time (≥ 4 h/day), and its combination with computer, tablet, or cell phone use (≥ 4 h/day), as well as sociodemographic, behavioral, and health characteristics were self-reported. Adjusted logistic regression was used to estimate odds ratios and 95% confidence intervals (95%CI). The prevalence of high sedentary behavior almost tripled when TV viewing was added to the time spent using other electronic devices (from 12.2%; 95%CI: 11.6; 12.8, to 34.7%; 95%CI: 33.8; 35.6), notably among the youngest (32.0 percentage points). Individuals living without a partner, who smoked, consumed alcohol and processed foods excessively, were physically inactive, and had hypertension were more likely to have both outcomes than their counterparts. Older and less educated individuals were more likely to spend excessive time watching TV and less likely to have high use of other electronic devices in addition to TV viewing than their peers. Including computer, tablet, or cell phone led to an increase in the prevalence of high sedentary behavior. The magnitude and direction of the associations of age and education with high sedentary behavior varied according to the method how high sedentary behavior was defined. Projects, programs, and policies must consider the different indicators of sedentary behavior in monitoring and promoting a healthier lifestyle.

**Supplementary Information:**

The online version contains supplementary material available at 10.1186/s12889-023-16517-7.

## Introduction

The growing interest in the study of sedentary behavior is justified by a notorious body of evidence [1, 2] showing its increasing presence in people’s daily lives, particularly in leisure time [3]. The results related to excessive time in sedentary behavior are of concern for public health, indicating an increased risk of mortality from all causes, including diabetes, cancer, and cardiovascular diseases, especially among physically inactive individuals [4–7].

Although TV time remains one of the most prevalent components of sedentary behavior in the adult population [8], the use of computers, tablets, or cell phones has been demonstrated in studies related to the subject because they are modern technologies [2] that can serve the same purpose as TVs [9]. A time trend study conducted in the United States found that the estimated prevalence of watching TV or videos remained high and stable from 2001 to 2016, while the prevalence of computer use during leisure time and total sitting time increased over the years among adults [10]. Similar trends were also observed in Australia among both men and women [8]. In Brazil, a study indicated different results for TV time, with a reduced trend of hours per day. However, there was an increased trend regarding other types of screen time (computer, tablet or smartphone use and playing videogames) [11].

As demonstrated in previous research, the sociodemographic correlates of sedentary time vary according to domains and indicators of sedentary behavior. For example, a systematic review found positive relationships between full-time employment with more time on passive commuting and less time on sedentary leisure-time behavior [12]. Living in more urban areas was associated with longer sitting times and total sedentary behavior, and those in more active work positions were more likely to have low occupational sedentary behavior [12]. Owning TVs also appeared to be a risk factor for more sedentary leisure-time behavior [12]. In Brazil, TV time, sedentary time spent on commuting, computer use at home, and time sitting at work were associated with different sociodemographic factors, in particular age, education, and socioeconomic status, showing different directions depending on the domains and indicators analyzed [13]. More recently, a study with Brazilian adults sought to investigate the correlates of different types of screen-based behavior [14]. The authors identified that the levels of sedentary behavior assessed by the time spent watching TV and the time using other electronic devices (computer, tablet, and cell phone) separately varied according to geographic, sociodemographic, behavioral, and health status characteristics.

In spite the correlates and health effects of TV viewing are well established [15], less is known about the potential sociodemographic correlates of other electronic devices, such as computers, tablets, and cell phones. Although the literature brings information about these different indicators of sedentary behavior [14], most of the previous studies have assessed the correlates of TV time and other devices combining these two indicators into one single variable [16, 17], which may provide a biased view on which population groups are more likely to engage in these behaviors. Moreover, because of low energy expenditure, the use of computer, tablet, or cell phone may also have adverse effects on health, even if in different magnitudes compared to watching TV [9]. On the other hand, each type of sedentary behavior and the factors that influence them may predispose people to different health outcomes [9]. Hence, the time using television and using other resources, such as computer, tablet or cell phone, if analyzed in isolation, may also not reflect the current pattern of sedentary behavior since they tend to involve different interests among people who use them [7, 9].

Considering that sedentary behavior is still the most common behavior performed during waking hours [18, 19], it is necessary to identify and understand the factors that influence the use of a single indicator and, simultaneously, the detailed correlates of the combination of this same indicator with others, as they coexist. Thus, taking into account that TV time, among all types of sedentary behavior, remains a classic behavior and that it is still the most known risk factor for all-cause mortality [4, 7, 20] – bringing a high concern for public health, the idea of observing it together with other screen time indicators allows us to verify whether this combination can differently influence the strength and direction of the association. The isolated and combined view of these behaviors can help to promote policies and strategies that aim to improve sedentary behavior more effectively, identifying priority groups. This information can be used to assist in the development of different interventions, focusing, for example, on education and awareness, counseling, community engagement, changes in the home environment and other contexts. Finally, as the 24 h of a day are made up of different movement patterns, replacing sedentary behavior during leisure time with other activities can have beneficial consequences for health, even with lighter intensity physical activities. Proposing changes related to sedentary behavior at work or commuting can often be unfeasible. In this perspective, the leisure domain allows more easily the engagement of practices to reduce this behavior. Therefore, this study aimed to investigate the consequences of including the time spent using electronic devices (computer, tablet, or cell phone) instead of considering only the TV time in the prevalence and factors associated with sedentary behavior among adults living in the capitals of the 26 Brazilian states and Federal District.

## Methods

### Study design and participants

This cross-sectional study used data from the Surveillance System for Risk and Protective Factors for Chronic Diseases by Telephone Survey (Vigitel) conducted in 2019. The survey was representative of the population aged at least 18 years, living in the 26 Brazilian state capitals and the Federal District, who had at least one fixed telephone line in their homes. The sample size calculation was based on parameters and estimates of an outcome prevalence of 50%, a coefficient of 95%, and a maximum error of around two percentage points, establishing a minimum sample size of two thousand individuals in each municipality investigated. Complementary information on the methodological aspects may be found in a previously published report [21].

### Outcome variables

As study outcomes, we considered: a) TV time and b) TV time plus time using computer, tablet, or cell phone. These sedentary behavior indicators performed during leisure time were obtained from the following questions: “On average, how many hours a day do you usually spend watching TV?”; “In your free time, do you usually use a computer, tablet, or cell phone to participate in social networks such as Facebook, watch movies, or play games?”; and “On average, how many hours of your free time (excluding work) do you usually spend on a computer, tablet, or cell phone?”. For each variable, high sedentary time was defined as ≥ 4 h/day. This threshold was based on previous research which showed negative results for cardiovascular diseases, mental disorders, and all-cause mortality [7, 22].

### Exposure variables

Five groups of sociodemographic and health characteristics were assessed: a) demographics indicators: such as sex (male and female), age (18 to 39, 40 to 59, and ≥ 60 years old), marital status (with and without a partner), skin color (white, black, brown, and others), regions of Brazil (North, Northeast, Central-West, Southeast, and South); b) social indicators: education level (0 to 4, 5 to 8, 9 to 11, and ≥ 12 years of study); c) lifestyle factors: smoking status (non-smoker, former smoker, or current smoker), excessive alcohol consumption (≥ 5 drinks for men and ≥ 4 drinks for women), consumption of processed foods (≥ 5 processed foods the day before the survey), performing physical activity during leisure time (yes or no), and meeting the physical activity recommendations in general(≥ 150 min/week of moderate physical activity, ≥ 75 min/week of vigorous physical activity, or an equivalent combination of moderate- and vigorous-intensity physical activity, considering the total time of physical activity in the leisure, commuting, and work domains); d) health condition aspects: presence of obesity, diabetes, and hypertension; and e) self-perceived health (very good/good, regular, bad/very bad).

### Statistical analysis

Data analysis considered the weighting scheme of the Vigitel survey, which aims to match the sociodemographic composition of the sample to the estimated composition of the total population of each city [21]. The Rake method was used to produce these estimates [23]. The statistical analysis included absolute and relative frequencies (%) and their respective 95% confidence intervals (95%CI). Crude and adjusted binary logistic regressions were used, with results expressed in odds ratios (OR). Results were deemed significant when *p* ≤ 0.05 according to the Wald heterogeneity test for nominal categorical variables and the linear trend test for ordinal categorical variables. For statistical modeling, we adopted the “backward” selection strategy and a critical level of *p* ≤ 0.20 to remain in the model to control confusion. In the analysis model, the variables were adjusted hierarchically at five levels: a) demographic variables; b) social factors; c) lifestyle aspects; d) health conditions; e) perception of health. The effects of each indicator on the outcomes were adjusted for other variables at the same level or higher. The software Stata® version 15.0 (Stata Corporation, College Station, United States) was the statistical package used for data analysis.

## Results

The analytical sample included 52,443 adults (response rate of 69.2%). The sample consisted predominantly of women (54%), and the mean age was 42.7 ± 14.3 years. The prevalence of sedentary behavior was 12.2% (95%CI: 11.6; 12.8) considering only excessive TV watching and 34.7% (95%CI: 33.8; 35.6) when combined with the use of computer, tablet, or cell phone.

Table 1 shows the prevalence and factors associated with TV time and its combination with the computer, tablet, or cell phone time. After the adjustment, it was found that older individuals, without a partner, with black skin color, smokers, that had high alcohol and processed foods consumption, who did not perform physical activities during their leisure time and did not meet the recommendations, and were diagnosed with hypertension were more exposed to excessive TV watching than their peers. Residents of the Central-West and South regions of Brazil and those with higher education level were less likely to watch TV for ≥ 4 h/day, in relation to those who lived in the North region and were less educated, respectively. When using other screen devices (computer, tablet or cell phone) was combined with watching TV, the associations remained in individuals without a partner, smokers, who drank and consumed processed foods in excess, did not perform physical activities during their leisure time and did not meet physical activity recommendations, had a diagnosis of hypertension, and lived in the Central-West and South regions of Brazil. In contrast, the associations for age and education level were inverted, indicating a protective factor for the elderly and a risk factor for the most educated people.

**Table 1:** Prevalence and factors associated with TV time and its combination with computer, tablet, or cell phone time among adults living in 26 Brazilian state capitals and the Federal District, 2019 (*n* = 52,443)

Variables	Television							Television + computer, tablet or cell phone						
	**%**^**d**^	**Crude**			**Adjusted**			**%**^**d**^	**Crude**			**Adjusted**		
		**OR**	**95% CI**	***p*-value**	**OR**	**95% CI**	***p*-value**		**OR**	**95% CI**	***p*-value**	**OR**	**95% CI**	***p*-value**
**Sex**				0.067^e^			0.621^e^				0.108^e^			0.437^e^
Male	11.5	1.00			1.00			35.5	1.00			1.00		
Female	12.7	1.12	0.99; 1.25		1.03	0.91; 1.16		34.0	0.93	0.86; 1.02		0.97	0.89; 1.05	
**Age (years)**				< 0.001^f^			< 0.001^f^				< 0.001^f^			< 0.001^f^
18 to 39	9.8	1.00			1.00			43.9	1.00			1.00		
40 to 59	12.1	1.27	1.10; 1.47		1.42	1.22; 1.66		26.8	0.47	0.43; 0.51		0.56	0.51; 0.62	
≥ 60	18.4	2.08	1.83; 2.37		2.27	1.95; 2.63		26.0	0.45	0.41; 0.49		0.53	0.48; 0.58	
**Marital status**				0.010^e^			< 0.001^e^				< 0.001^e^			< 0.001^e^
With a partner	11.3	1.00			1.00			26.1	1.00			1.00		
Without a partner	12.9	1.16	1.04; 1.30		1.38	1.22; 1.56		42.3	2.08	1.91; 2.26		1.79	1.65; 1.95	
**Skin color**				< 0.001^e^			0.002^e^				< 0.001^e^			0.178^e^
White	11.6	1.00			1.00			34.0	1.00			1.00		
Black	14.6	1.30	1.06; 1.59		1.36	1.10; 1.68		39.8	1.28	1.11; 1.48		1.12	0.97; 1.30	
Brown	11.3	0.97	0.85; 1.10		1.02	0.89; 1.16		35.3	1.05	0.96; 1.16		0.98	0.89; 1.08	
Others	16.4	1.49	1.22; 1.82		1.35	1.09; 1.66		29.8	0.82	0.70; 0.96		0.91	0.78; 1.07	
**Regions of Brazil**				< 0.001^e^			< 0.001^e^				< 0.001^e^			< 0.001^e^
North	11.7	1.00			1.00			36.8	1.00			1.00		
Northeast	12.5	1.09	0.95; 1.24		1.02	0.89; 1.17		35.1	0.93	0.85; 1.01		0.94	0.85; 1.03	
Central-West	9.7	0.81	0.67; 0.97		0.79	0.65; 0.95		31.2	0.78	0.69; 0.87		0.80	0.71; 0.91	
Southeast	13.1	1.15	0.98; 1.33		1.08	0.92; 1.27		35.9	0.96	0.86; 1.07		1.05	0.94; 1.18	
South	9.7	0.82	0.69; 0.96		0.78	0.66; 0.93		29.5	0.72	0.64; 0.81		0.77	0.68; 0.88	
**Education (years of study)**				< 0.001^f^			< 0.001^f^				< 0.001^f^			< 0.001^f^
0 to 4	15.6	1.00			1.00			20.9	1.00			1.00		
5 to 8	15.7	1.00	0.84; 1.21		1.18	0.98; 1.42		28.5	1.51	1.28; 1.78		1.36	1.14; 1.61	
9 to 11	13.8	0.86	0.74; 1.00		1.15	0.98; 1.35		40.7	2.60	2.28; 2.96		1.88	1.62; 2.18	
≥ 12	7.2	0.42	0.35; 0.50		0.56	0.47; 0.68		36.1	2.14	1.87; 2.45		1.47	1.26; 1.72	
**Smoking status**				< 0.001^e^			< 0.001^e^				< 0.001^e^			< 0.001^e^
Non-smoker	10.8	1.00			1.00			34.4	1.00			1.00		
Former smoker	13.8	1.32	1.16; 1.50		1.09	0.94; 1.25		31.8	0.89	0.80; 0.98		1.11	0.99; 1.24	
Smoker	18.9	1.93	1.61; 2.31		1.53	1.26; 1.86		42.8	1.43	1.23; 1.66		1.36	1.16; 1.59	
**Excessive alcohol consumption**^**a**^				0.010^e^			< 0.001^e^				< 0.001^e^			< 0.001^e^
No	11.7	1.00			1.00			31.8	1.00			1.00		
Yes	14.0	1.22	1.05; 1.43		1.36	1.15; 1.60		47.4	1.94	1.74; 2.16		1.55	1.39; 1.74	
**Processed foods**^**b**^				0.018^e^			0.001^e^				< 0.001^e^			< 0.001^e^
< 4 processed foods	11.8	1.00			1.00			32.4	1.00			1.00		
≥ 5 processed foods	13.9	1.21	1.03; 1.43		1.32	1.12; 1.55		45.2	1.72	1.54; 1.92		1.46	1.30; 1.64	
**Leisure time physical activity**				< 0.001^e^			< 0.001^e^				0.003^e^			0.009^e^
Yes	9.3	1.00			1.00			33.3	1.00			1.00		
No	15.3	1.75	1.56; 1.96		1.31	1.14; 1.50		36.2	1.13	1.05; 1.23		1.14	1.03; 1.27	
**Physical activity recommendations**^**c**^				< 0.001^e^			< 0.001^e^				0.002^e^			< 0.001^e^
Yes	9.5	1.00			1.00			33.4	1.00			1.00		
No	15.4	1.72	1.53; 1.93		1.34	1.17; 1.53		36.3	1.14	1.05; 1.23		1.26	1.14; 1.39	
**Obesity**				0.001^e^			0.200^e^				0.469^e^			0.839^e^
No	11.4	1.00			1.00			35.2	1.00			1.00		
Yes	14.0	1.26	1.10; 1.44		1.09	0.95; 1.25		34.3	0.96	0.87; 1.07		0.99	0.89; 1.10	
**Diabetes**				< 0.001^e^			0.072^e^				< 0.001^e^			0.193^e^
No	11.6	1.00			1.00			35.2	1.00			1.00		
Yes	19.1	1.81	1.56; 2.09		1.17	0.99; 1.40		28.8	0.75	0.66; 0.85		1.10	0.95; 1.26	
**Arterial hypertension**				< 0.001^e^			< 0.001^e^				< 0.001^e^			0.004^e^
No	10.7	1.00			1.00			36.1	1.00			1.00		
Yes	16.6	1.67	1.49; 1.87		1.33	1.16; 1.54		30.4	0.78	0.71; 0.85		1.16	1.05; 1.29	
**Self-perceived health**				< 0.001^f^			0.072^f^				0.001^f^			0.002^f^
Very good / Good	10.7	1.00			1.00			33.6	1.00			1.00		
Regular	14.5	1.42	1.26; 1.60		1.09	0.94; 1.26		37.2	1.17	1.07; 1.28		1.18	1.07; 1.29	
Bad / Very bad	17.3	1.75	1.42; 2.14		1.25	0.97; 1.59		36.3	1.13	0.95; 1.34		1.15	0.95; 1.39	

^a^excessive alcohol consumption, considering ≥ 5 drinks for males and ≥ 4 drinks for females

^b^consumption of processed foods the day before the survey

^c^the meeting of physical activity recommendations considers ≥ 150 min per week of moderate physical activity, ≥ 75 min per week of vigorous physical activity, or an equivalent combination of moderate and vigorous physical activity

^d^weighted percentage of the sample that spends at least four hours on the investigated outcomes; OR: odds ratio; 95% CI: 95% confidence interval

^e^*p*-value from the Wald heterogeneity test

^f^*p*-value from the Wald test for linear trend; adjusted analysis for sex, age, marital status, skin color, and region of Brazil (first level); education level (second level); smoking, alcohol consumption, processed foods, leisure-time physical activity, and physical activity recommendations (third level); obesity, diabetes, and arterial hypertension (fourth level); self-perceived health (fifth level)

The difference in percentage points (pp) of the prevalence of sedentary behavior when considering only the excessive time watching TV and when combining it with the time spent using other electronic devices according to the sociodemographic and lifestyle characteristics, health conditions, and perception of health of the participants is shown in Table 2. Overall, all characteristics showed a statistically significant increase in the prevalence of sedentary behavior by incorporating the time spent on other electronic devices in addition to the TV time. This is marked in younger individuals (32.0 pp), those who consumed alcohol (27.7 pp) and processed foods (26.6 pp) in excess, lived without a partner (26.3 pp), and had higher education levels (25.5 pp).

**Table 2:** Difference in prevalence of sedentary behavior considering only the time spent watching television and its combination with time spent using computers, tablets, or cell phones among adults living in the 26 capitals of Brazilian states and the Federal District, 2019 (*n* = 52,443)

Variables	Crude		Adjusted	
	**∂**	**95% CI**	**∂**	**95% CI**
**Sex**				
Male	24.0	22.6; 25.4	23.1	21.9; 24.4
Female	21.3	20.3; 22.4	22.3	21.2; 23.3
**Age (years)**				
18 to 39	34.1	32.6; 35.7	32.0	30.5; 33.6
40 to 59	14.6	13.6; 15.7	15.9	14.8; 17.0
≥ 60	7.7	7.0; 8.4	8.9	7.6; 9.2
**Marital status**				
With a partner	14.8	13.8; 15.8	17.6	16.4; 18.7
Without a partner	29.4	28.1; 30.7	26.3	25.1; 27.4
**Skin color**				
White	22.4	21.1; 23.8	23.5	22.1; 24.8
Black	25.2	22.4; 27.9	22.6	20.2; 25.0
Brown	24.0	22.6; 25.3	22.9	21.6; 24.2
Others	13.4	11.0; 15.7	16.9	14.2; 19.6
**Regions of Brazil**				
North	25.1	23.6; 26.7	23.5	22.0; 24.9
Northeast	22.5	21.5; 23.6	21.9	20.9; 22.9
Central-West	21.5	19.8; 23.2	21.3	19.6; 22.9
Southeast	22.8	21.1; 24.4	23.7	22.0; 25.4
South	19.8	18.1; 21.5	20.4	18.6; 22.2
**Education (years of study)**				
0 to 4	5.3	4.0; 6.5	9.2	7.0; 11.4
5 to 8	12.8	10.9; 14.7	16.8	14.4; 19.1
9 to 11	26.9	25.5; 28.3	24.9	23.6; 26.1
≥ 12	28.9	27.3; 30.5	25.5	24.1; 26.9
**Smoking status**				
Non-smoker	23.6	22.6; 24.6	22.3	21.3; 23.2
Former smoker	18.0	16.3; 19.8	23.8	21.8; 25.9
Smoker	24.0	20.9; 27.1	24.1	21.2; 27.0
**Excessive alcohol consumption**^a^				
No	20.0	19.2; 20.9	21.3	20.4; 22.2
Yes	33.4	31.1; 35.8	27.7	25.7; 29.7
**Processed foods**^b^				
< 4 processed foods	20.6	19.7; 21.5	21.7	20.8; 22.6
≥ 5 processed foods	31.3	28.9; 33.6	26.6	24.6; 28.5
**Leisure time physical activity**				
Yes	24.0	22.8; 25.2	22.5	21.3; 23.7
No	20.9	19.8; 22.1	22.9	21.5; 24.2
**Physical activity recommendations**^c^				
Yes	23.8	22.6; 25.0	22.0	21.0; 23.1
No	21.0	19.8; 22.1	23.6	22.4; 24.8
**Obesity**				
No	23.8	22.8; 24.8	23.4	22.4; 24.3
Yes	20.4	18.6; 22.2	22.6	20.7; 24.5
**Diabetes**				
No	23.6	22.7; 24.5	22.8	22.0; 23.7
Yes	9.8	8.1; 11.4	19.8	16.8; 22.8
**Arterial hypertension**				
No	25.4	24.4; 26.4	23.1	22.2; 24.0
Yes	13.8	12.5; 15.1	23.8	21.7; 26.0
**Self-perceived health**				
Very good / Good	22.9	21.9; 24.0	22.2	21.2; 23.1
Regular	22.7	21.1; 24.2	24.5	22.9; 26.1
Bad / Very bad	19.0	15.9; 22.2	22.4	18.9; 25.9

^a^excessive alcohol consumption, considering ≥ 5 drinks for males and ≥ 4 drinks for females

^b^consumption of processed foods the day before the survey

^c^the meeting of physical activity recommendations considers ≥ 150 min per week of moderate physical activity, ≥ 75 min per week of vigorous physical activity, or an equivalent combination of moderate and vigorous physical activity; ∂: difference in percentage points; 95% CI: 95% confidence interval

**Fig. 1:**
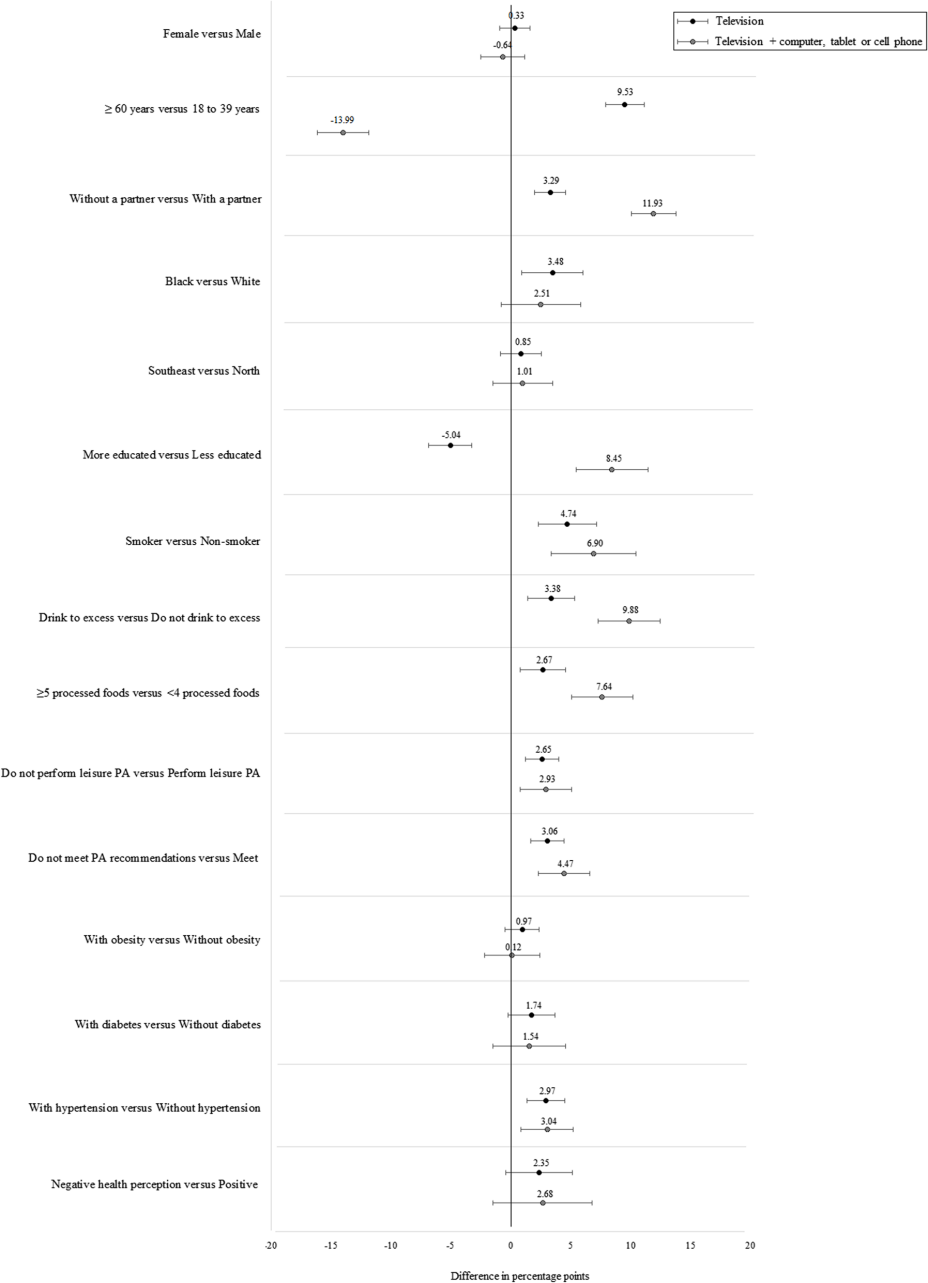
Magnitude of the associations between exposure variables and TV viewing and its combination with other screens. Notes: The magnitude of the associations is represented by differences in percentage points between extreme categories of investigated exposures. The results of the adjusted analysis controlled for demographic, social, lifestyle, and health conditions (related to the presence of the investigated chronic diseases) and self-perceived health variables of 52,443 residents of the 26 capitals of Brazilian states and the Federal District, 2019

Figure 1 shows the comparison between the prevalence of sedentary behavior based only on the time spent watching TV and its combination with computer, tablet, or cell phone use among the extreme categories of the characteristics investigated. While lifestyle variables were associated with greater use of both outcomes, the age and education level showed associations in opposite directions when considering computer, tablet, or cell phone use.

## Discussion

This study aimed to investigate the consequences of including the time spent using electronic devices (computer, tablet or cell phone) to the leisure time spent watching TV when determining the prevalence and factors associated with sedentary behavior in a representative sample of adults living in the capitals of the 26 Brazilian states and the Federal District. The time spent on various electronic devices considerably increased the prevalence of excessive sedentary behavior. Furthermore, behavioral variables were strongly associated with sedentary behavior, regardless of the investigated outcome. The color of the skin was associated only with TV time, while the self-perception of health was associated with the combination of different types of sedentary behavior. However, for age and education level, associations were observed in opposite directions when only excessive TV time was considered and in combination with computer, tablet or cell phone use.

The rapid technological advancement, coupled with the new habits of modern society (related to urbanization and food, transport, and economic systems), in addition to the change in interpersonal relationships contributed to reducing human movement and to the increasingly frequent and prolonged use of electronic equipment [24–26]. This scenario may be observed from the results of the present study when considering the TV time alone and combined with the use of computer, tablet or cell phone, in which the prevalence of sedentary behaviors almost triples (from 12.2% to 34.7%).

One of the most interesting results of this study concerns the variables associated differently depending on the screen-based behaviors considered to determine sedentary behavior. Excessive TV viewing was associated with older individuals and those with lower education levels. In turn, when considering other electronic devices, these associations were inverted, and sedentary behavior was higher among younger and more educated. The age-related results are consistent with the literature, which states that older individuals tend to spend more time watching TV [27], while younger individuals opt for other screen-based behaviors, such as using computer and cell phone [28]. The advent of technology has a very likely relationship with this result since younger individuals enjoy more and handle more easily innovations in information technology, through increasingly sophisticated and multifunctional equipment, while the advancement of age is a reason for difficulty, besides the resistance to new developments in this market [29]. From a different point of view, the level of education has been shown to be an important variable in epidemiological studies since it is simple and reliable to collected, in addition to maintaining an important relationship with income, type of work, and the living conditions of the population [30]. Thus, it is hypothesized that increased purchasing power and the consumption of technologies by subjects with a more extensive educational background may explain the higher prevalence of sedentary behavior in leisure time when the different electronic devices investigated were taken into account. The results of the present study, which compared the extreme categories of age and education, pointed to even more discrepant results, which confirms the hypotheses previously raised.

For the skin color, an association was only observed when considering the TV time alone. Few studies have found associations between skin color and sedentary behavior, especially during leisure time. When considering the commuting domain, Cohen et al. [31] found that black people spend more time sitting in the car or on the bus than white people, while, in the work domain, this association seems to be inverse, with white people being more likely to remain in sedentary behavior, in this case using computer. Thus, factors such as social and economic aspects seem to be directly associated with the time spent in sedentary activities [32], so skin color in itself is not a determinant factor for assessing sedentary behavior.

In contrast, the self-perception of health was only associated when TV time was combined with the use of computers, tablets, or cell phones, indicating that the more negative the individual perceived their health status, the greater the chance of using such devices for ≥ 4 h/day. Gaskin and Orellana [16] also found similar results, although they considered the total time spent in sedentary behaviors (sitting or reclining) in a typical day and not just during leisure time. For the authors, self-reported health may correspond to other aspects beyond the diagnosis of diseases and that are often not identified by questions asked by questionnaires or interviews [16], with the participant having a broader view of health in general. From another perspective, a study developed in South Korea found that lower levels of emotional self-perception and general health status were associated with excessive smartphone use [33] and that such people may be trapped in a vicious cycle. Alhassan et al. [34] explained that, due to the stress caused by a certain emotional issue or general health, people try to compensate or overcome such feelings with the excessive use of smartphones, without realizing that this addiction also has a negative impact on health and social, emotional and physical well-being.

Individuals without partners were more likely to have both investigated outcomes. These findings are likely to be related to the fact that single, separated, and widowed people use both TV and other devices as a form of social interaction. The diversity of content, including sporting events such as the Olympic Games and the FIFA World Cup, allows people to interact and reinforces the power of these screen-based indicators as the main means of trying to explore varied experiences through social networking services [35]. Moreover, it is evident that the use of mobile devices has become increasingly present in people's daily lives, especially when connected to the Internet [36]. Research carried out in 27 countries detected that 83% of Brazilian adults had a cell phone, with the majority (60%) being smartphone models [37]. Thus, given the easy access to social media and other related resources, those without partners are able to interact more, while those who live with someone and have an established family routine are less likely to use these indicators of sedentary behavior.

The geographic region also seems to be a determining factor for the scenario investigated, with similar associations when considering only the time spent watching TV and its combination with that spent on computers, tablets, or cell phones. Studies indicate that the perception of security in environments near homes, for example, may contribute to the performance or not of external activities [38, 39]; in some cases, people are more afraid to leave their homes and, thus, make more use of screen devices. Therefore, public policies which aim at creating safe spaces, leisure and cultural options, as well as opportunities to choose in one’s free time, seem to be essential for a more active city, where people can replace time spent in sedentary activities with healthier behaviors. Even so, it is important to recognize the existence of some barriers to performing more active practices during leisure time, such as lack of motivation and time [40], allowing more time to use technologies in this context.

Being a smoker and consuming alcohol and processed foods in excess, not performing physical activity during leisure time, and not meeting physical activity recommendations were identified as risk factors for both outcomes of this study. These findings suggest that there may be a tendency for people who engage in risky health behaviors to acquire other risky behaviors [41]. The release of hormones that stimulate the feeling of well-being and, from another perspective, one’s motivational levels may also be related to the consumption of cigarettes, alcohol, and/or processed foods, in addition to the increase in time spent in sedentary behaviors and other metabolic indicators which are unfavorable to health and well-being [17, 42, 43]. Thereby, when dealing with a leisure context, in which the individual is usually relaxed, they start to consume these types of products more.

Human movement behaviors have different intensities, varying on a continuum which includes sleep, sedentary behaviors, and light, moderate, or vigorous physical activities [44]. Thus, it is not possible to state that an individual who spends substantial amount of time in sedentary behaviors will not be able to meet the physical activity recommendations [45], considering that these behaviors can be accumulated over the 24 h of the day and, consequently, coexist [46]. Further studies are therefore needed to delve deeper into the interaction mechanism between physical inactivity and sedentary behavior to propose strategies that contribute to the promotion of healthier lifestyles [47]. This becomes relevant because these behaviors, when analyzed together, may be contributors to the increase in cases of non-communicable chronic diseases [47, 48].

In this study, among the variables related to health conditions, only arterial hypertension was associated with the investigated outcomes. Research conducted with a multiethnic Asian population has shown that TV viewing was associated with increased systolic blood pressure and other health indicators such as high total cholesterol, triglycerides, and C-reactive protein levels, insulin resistance, and lower adiponectin levels [49]. However, the authors did not find associations between reading time and computer use with these biomarkers, which are directly related to the diagnosis of the mentioned chronic diseases [49]. Although older, another study involving only American women found that the time spent sitting at work and watching TV was associated with the risk of diabetes and obesity, but the time sitting at home during leisure, used for reading, meals, and among other activities, was associated only with diabetes [50]. A possible explanation for such inconsistencies may be related to the types of sedentary behavior, which have been classified primarily according to the cognitive effort required for the activities. Evidence shows that “mentally passive” sedentary behaviors, such as spending time watching TV or sitting while listening to music, may be more harmful to health than “mentally active” behaviors, exemplified by computer use, reading books or newspapers, and participating in meetings [51, 52].

Still regarding the associations between chronic diseases and sedentary behavior, the domains of physical activity and the presence of multimorbidity can also contribute to these results. A study developed by Araujo et al. [53] in Brazilian adults analyzed the associations between TV viewing and other screens with obesity, diabetes and hypertension and the moderating role of physical activity domains in these associations. Being physically active in occupational context reduced the association between TV viewing and hypertension, but increased the associations between others indicators of screen time with obesity and hypertension [53]. On the other hand, not meeting leisure physical activity recommendations increased the association between other screens and obesity and hypertension [53]. Another study carried out in Brazil, which aimed to verify the association between TV viewing and other types of screens with multimorbidity, found that only the habit of watching TV was associated with the outcome of interest, while other types of screens were associated with multimorbidity only in some specific cases, depending on the time categories between men and elderly [54]. Although in both studies sedentary behavior was observed as an exposure variable and chronic diseases, as outcomes the findings allow us to reflect on the importance of taking into account the domains of physical activity and the presence of multimorbidity in analysis involving sedentary behavior. In the present study, only the total and the leisure physical activity recommendations were used as adjustment variables, as well as the presence of chronic diseases, analyzed individually.

As strengths and limitations of this study, the size and representativeness of the sample are highlighted. Concerning the content, it is noteworthy that the analysis of sedentary behavior went beyond the investigation of isolated TV time, considering the inclusion of the time spent on other electronic devices currently being made evident in the literature. As it involved a telephone survey, it was not possible to use objective measures such as accelerometers, but the use of a questionnaire made it possible to understand different indicators of sedentary behavior, although complementary information, such as the purpose or the contents accessed by participants, were not explored. Unfortunately, the sedentary behavior variables from the Vigitel survey were not collected continuously. Participants had eight response options for these questions: a) people who do not watch television or do not use computer, tablet or cell phone, b) people who spend less than one hour a day in sedentary behavior; c) people who spend between 1 and 2 two hours a day; d) 2 to 3 h; e) 3 to 4 h; f) 4 to 5 h; g) 5 to 6 h; h) 6 h or more. Thus, a Supplementary material is available with a multinomial regression, in which it is possible to analyze the factors associated with the outcomes, categorized as follows: a) ≤ 2 h/day; b) 3 to 4 h/day; c) ≥ 5 h/day. It is noteworthy that, in general, the results were quite similar to those of the binary logistic regression. Another aspect related to the outcome variables is that both only accounted for the time spent using screen-based devices during leisure, disregarding other domains. In contrast, the amount of research on sedentary behaviors in this context is reiterated because of the risks caused by them, as well as the greater possibility of interventions developed in free time [55]. Finally, the results founded should be interpreted with caution due to the cross-sectional design, since it is not possible to attribute causality between outcomes and the variables investigated.

## Conclusions

Even though the cut-off point for both investigated outcomes remained the same, the use of different electronic devices during leisure modifies the prevalence and factors associated with sedentary behavior exclusively derived from excessive TV viewing among Brazilian adults. Future studies should examine the combination of other types of sedentary behavior and investigate other domains besides leisure, given that most of the day is made up of activities of this nature. Additionally, it is suggested that analyses be carried out considering the differentiation between active and passive sedentary behaviors. Finally, projects, programs, and policies must consider the different indicators of sedentary behavior in monitoring and promoting a healthier lifestyle.

### Supplementary Information

**Additional file 1. **

## Data Availability

The datasets used and/or analyzed during the current study available from the corresponding author on reasonable request.

## Abbreviations

TV: Television
95% CI: 95% confidence intervals
OR: Odds ratios
