# Correction: Use of electronic devices in leisure time modifies the prevalence and factors associated with sedentary behavior derived exclusively from excessive television viewing among Brazilian adults

**DOI:** 10.1186/s12889-023-16716-2

**Published:** 2023-09-18

**Authors:** Cecília Bertuol, Murilo Henrique Corrêa da Silveira, Rodrigo de Rosso Krug, Juliedy Waldow Kupske, Grégore Iven Mielke, Giovani Firpo Del Duca

**Affiliations:** 1 Graduate Program in Physical Education, Federal University of Santa Catarina, Campus Universitário Reitor João David Ferreira Lima, Florianópolis, SC 88040-900 Brazil; 2 Graduate Program in Integrative Health Care, University of Cruz Alta, Cruz Alta, RS 98020-290 Brazil; 3 Graduate Program in Human Movement Science, Federal University of Rio Grande Do Sul, Rua Felizardo 750, Porto Alegre, RS 90690-200 Brazil; 4 School of Public Health, The University of Queensland, Brisbane, QLD 4006 Australia

**Correction: BMC Public Health 23, 1602 (2023)**

**https://doi.org/10.1186/s12889-023-16517-7**

The original publication of this article [1] contained an incorrect version of Fig. 1. The corrected figure is shown in this correction article, the original article has been updated. The publisher apologizes for the inconvenience caused to the authors & readers.

**Fig. 1:**
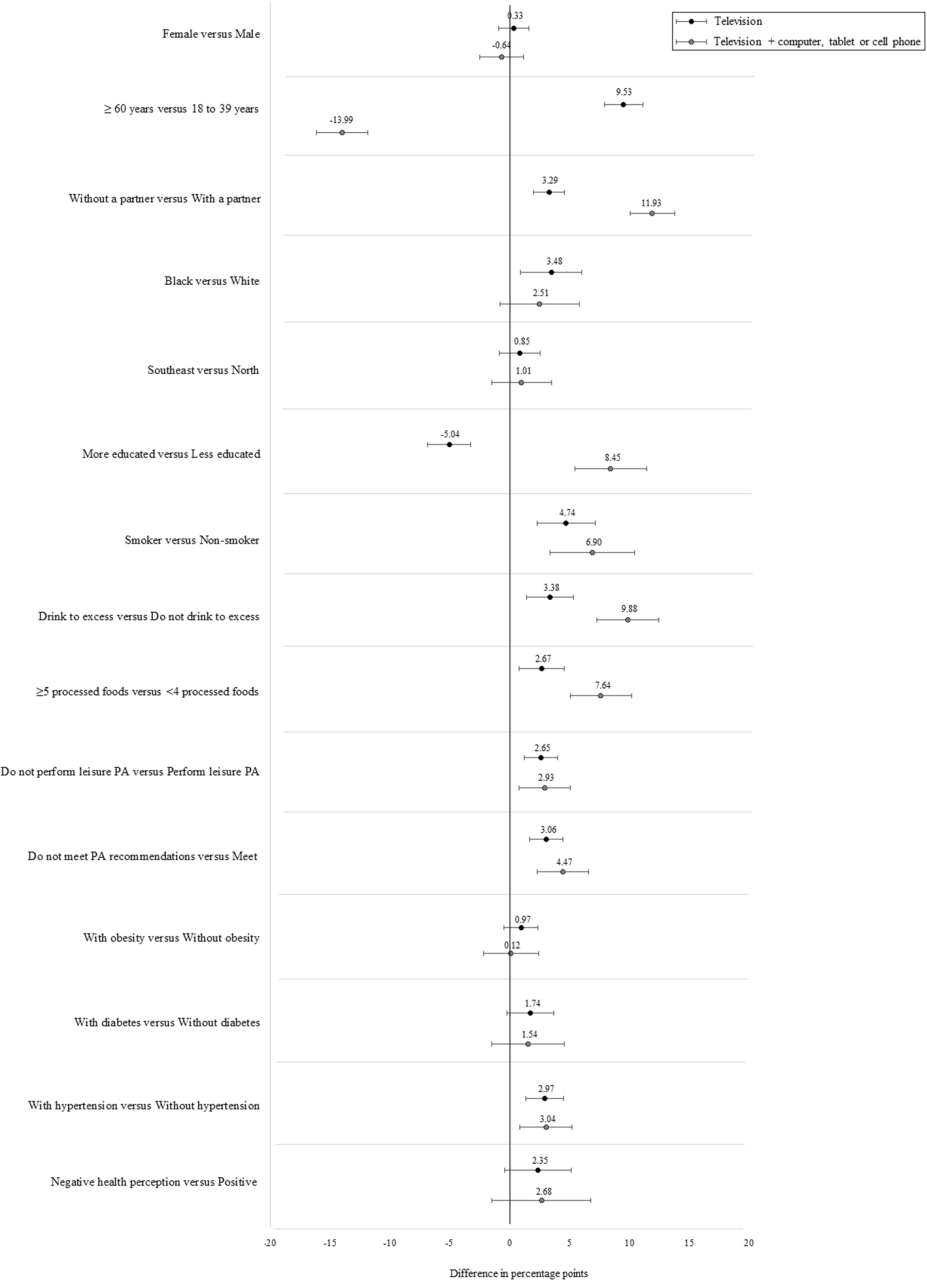
Magnitude of the associations between exposure variables and TV viewing and its combination with other screens. Notes: The magnitude of the associations is represented by differences in percentage points between extreme categories of investigated exposures. The results of the adjusted analysis controlled for demographic, social, lifestyle, and health conditions (related to the presence of the investigated chronic diseases) and self-perceived health variables of 52,443 residents of the 26 capitals of Brazilian states and the Federal District, 2019
