# Supplementary material for: Use of electronic devices in leisure time modifies the prevalence and factors associated with sedentary behavior derived exclusively from excessive television viewing among Brazilian adults
Source: BMC Public Health. 2023 Aug 23;23:1602. doi: 10.1186/s12889-023-16517-7 (PMC10463304; doi:10.1186/s12889-023-16517-7)
Supplement: Supplementary file 1 — Additional file 1. [file 12889_2023_16517_MOESM1_ESM.docx]

Table 1. Adjusted analyses of TV time and its combination with computer, tablet, or cell phone time among adults living in 26 Brazilian state capitals and the Federal District, 2019 (n = 52,443).

| **Variables** | **Television** | | | | | | |  | **Television + computer, tablet or cell phone** | | | | | | |
| --- | --- | --- | --- | --- | --- | --- | --- | --- | --- | --- | --- | --- | --- | --- | --- |
|  | **3 to 4 hours x ≤ 2 hours** | | |  | **≥ 5 hours x ≤ 2 hours** | | |  | **3 to 4 hours x ≤ 2 hours** | | |  | **≥ 5 hours x ≤ 2 hours** | | |
|  | **OR** | **95% CI** | **p-value** |  | **OR** | **95% CI** | **p-value** |  | **OR** | **95% CI** | **p-value** |  | **OR** | **95% CI** | **p-value** |
| **Sex** |  |  | 0.587 |  |  |  | 0.076 |  |  |  | 0.014 |  |  |  | 0.454 |
| Male | 1.00 |  |  |  | 1.00 |  |  |  | 1.00 |  |  |  | 1.00 |  |  |
| Female | 0.97 | 0.87; 1.08 |  |  | 1.14 | 0.99; 1.33 |  |  | 0.89 | 0.81; 0.98 |  |  | 0.96 | 0.87; 1.06 |  |
| **Age (years)** |  |  | <0.001 |  |  |  | <0.001 |  |  |  | <0.001 |  |  |  | <0.001 |
| 18 to 39 | 1.00 |  |  |  | 1.00 |  |  |  | 1.00 |  |  |  | 1.00 |  |  |
| 40 to 59 | 1.38 | 1.21; 1.57 |  |  | 1.57 | 1.29; 1.91 |  |  | 0.68 | 0.60; 0.76 |  |  | 0.49 | 0.44; 0.55 |  |
| ≥ 60 | 1.87 | 1.65; 2.13 |  |  | 2.86 | 2.37; 3.44 |  |  | 0.64 | 0.57; 0.72 |  |  | 0.46 | 0.41; 0.52 |  |
| **Marital status** |  |  | <0.001 |  |  |  | <0.001 |  |  |  | <0.001 |  |  |  | <0.001 |
| With a partner | 1.00 |  |  |  | 1.00 |  |  |  | 1.00 |  |  |  | 1.00 |  |  |
| Without a partner | 1.23 | 1.11; 1.37 |  |  | 1.66 | 1.43; 1.92 |  |  | 1.43 | 1.30; 1.57 |  |  | 2.20 | 1.99; 2.44 |  |
| **Skin color** |  |  | 0.275 |  |  |  | 0.004 |  |  |  | 0.043 |  |  |  | 0.698 |
| White | 1.00 |  |  |  | 1.00 |  |  |  | 1.00 |  |  |  | 1.00 |  |  |
| Black | 1.05 | 0.86; 1.29 |  |  | 1.56 | 1.22; 1.99 |  |  | 0.86 | 0.72; 1.02 |  |  | 1.20 | 1.00; 1.43 |  |
| Brown | 1.06 | 0.93; 1.19 |  |  | 1.06 | 0.90; 1.25 |  |  | 0.96 | 0.87; 1.07 |  |  | 1.01 | 0.90; 1.13 |  |
| Others | 1.07 | 0.88; 1.30 |  |  | 1.70 | 1.31; 2.21 |  |  | 0.75 | 0.62; 0.89 |  |  | 0.92 | 0.76; 1.11 |  |
| **Regions of Brazil** |  |  | 0.031 |  |  |  | 0.470 |  |  |  | 0.084 |  |  |  | 0.630 |
| North | 1.00 |  |  |  | 1.00 |  |  |  | 1.00 |  |  |  | 1.00 |  |  |
| Northeast | 0.91 | 0.80; 1.03 |  |  | 0.97 | 0.81; 1.15 |  |  | 1.03 | 0.92; 1.14 |  |  | 0.94 | 0.84; 1.05 |  |
| Central-West | 0.84 | 0.71; 0.98 |  |  | 0.77 | 0.60; 0.98 |  |  | 0.95 | 0.83; 1.08 |  |  | 0.78 | 0.68; 0.90 |  |
| Southeast | 1.12 | 0.96; 1.30 |  |  | 1.00 | 0.82; 1.22 |  |  | 1.12 | 0.99; 1.28 |  |  | 1.08 | 0.95; 1.24 |  |
| South | 0.91 | 0.78; 1.07 |  |  | 0.69 | 0.55; 0.86 |  |  | 1.04 | 0.91; 1.20 |  |  | 0.76 | 0.65; 0.88 |  |
| **Education (years of study)** |  |  | 0.002 |  |  |  | <0.001 |  |  |  | <0.001 |  |  |  | 0.022 |
| 0 to 4 | 1.00 |  |  |  | 1.00 |  |  |  | 1.00 |  |  |  | 1.00 |  |  |
| 5 to 8 | 1.07 | 0.88; 1.30 |  |  | 1.13 | 0.92; 1.39 |  |  | 1.30 | 1.08; 1.57 |  |  | 1.40 | 1.15; 1.70 |  |
| 9 to 11 | 1.15 | 0.97; 1.36 |  |  | 0.97 | 0.80; 1.18 |  |  | 1.72 | 1.46; 2.02 |  |  | 2.07 | 1.75; 2.44 |  |
| ≥ 12 | 0.79 | 0.66; 0.95 |  |  | 0.38 | 0.30; 0.48 |  |  | 1.67 | 1.42; 1.98 |  |  | 1.38 | 1.16; 1.65 |  |
| **Smoking status** |  |  | <0.001 |  |  |  | <0.001 |  |  |  | 0.074 |  |  |  | <0.001 |
| Non-smoker | 1.00 |  |  |  | 1.00 |  |  |  | 1.00 |  |  |  | 1.00 |  |  |
| Former smoker | 1.16 | 1.01; 1.32 |  |  | 1.08 | 0.91; 1.27 |  |  | 1.10 | 0.97; 1.24 |  |  | 1.10 | 0.96; 1.26 |  |
| Smoker | 1.54 | 1.28; 1.85 |  |  | 1.63 | 1.29; 2.06 |  |  | 1.14 | 0.95; 1.37 |  |  | 1.41 | 1.17; 1.69 |  |
| **Excessive alcohol consumption^a^** |  |  | <0.001 |  |  |  | <0.001 |  |  |  | <0.001 |  |  |  | <0.001 |
| No | 1.00 |  |  |  | 1.00 |  |  |  | 1.00 |  |  |  | 1.00 |  |  |
| Yes | 1.37 | 1.18; 1.59 |  |  | 1.46 | 1.18; 1.81 |  |  | 1.51 | 1.32; 1.73 |  |  | 1.90 | 1.66; 2.18 |  |
| **Processed foods^b^** |  |  | 0.002 |  |  |  | 0.001 |  |  |  | 0.001 |  |  |  | <0.001 |
| < 4 processed foods | 1.00 |  |  |  | 1.00 |  |  |  | 1.00 |  |  |  | 1.00 |  |  |
| ≥ 5 processed foods | 1.27 | 1.09; 1.48 |  |  | 1.39 | 1.15; 1.70 |  |  | 1.25 | 1.09; 1.43 |  |  | 1.63 | 1.43; 1.86 |  |
| **Leisure time physical activity** |  |  | 0.318 |  |  |  | <0.001 |  |  |  | 0.847 |  |  |  | 0.012 |
| Yes | 1.00 |  |  |  | 1.00 |  |  |  | 1.00 |  |  |  | 1.00 |  |  |
| No | 1.07 | 0.94; 1.21 |  |  | 1.51 | 1.28; 1.78 |  |  | 1.01 | 0.90; 1.14 |  |  | 1.16 | 1.03; 1.31 |  |
| **Physical activity recommendations^c^** | |  | 0.012 |  |  |  | <0.001 |  |  |  | 0.051 |  |  |  | <0.001 |
| Yes | 1.00 |  |  |  | 1.00 |  |  |  | 1.00 |  |  |  | 1.00 |  |  |
| No | 1.17 | 1.04; 1.33 |  |  | 1.38 | 1.17; 1.63 |  |  | 1.13 | 1.00; 1.27 |  |  | 1.36 | 1.21; 1.53 |  |
| **Obesity** |  |  | 0.535 |  |  |  | 0.110 |  |  |  | 0.189 |  |  |  | 0.251 |
| No | 1.00 |  |  |  | 1.00 |  |  |  | 1.00 |  |  |  | 1.00 |  |  |
| Yes | 1.04 | 0.92; 1.18 |  |  | 1.15 | 0.97; 1.36 |  |  | 1.09 | 0.96; 1.23 |  |  | 1.08 | 0.95; 1.22 |  |
| **Diabetes** |  |  | 0.132 |  |  |  | 0.056 |  |  |  | 0.150 |  |  |  | 0.302 |
| No | 1.00 |  |  |  | 1.00 |  |  |  | 1.00 |  |  |  | 1.00 |  |  |
| Yes | 1.14 | 0.96; 1.37 |  |  | 1.22 | 0.99; 1.49 |  |  | 0.89 | 0.75; 1.04 |  |  | 1.10 | 0.92; 1.32 |  |
| **Arterial hypertension** |  |  | 0.001 |  |  |  | <0.001 |  |  |  | 0.693 |  |  |  | <0.001 |
| No | 1.00 |  |  |  | 1.00 |  |  |  | 1.00 |  |  |  | 1.00 |  |  |
| Yes | 1.27 | 1.10; 1.46 |  |  | 1.40 | 1.19; 1.64 |  |  | 1.02 | 0.91; 1.15 |  |  | 1.30 | 1.14; 1.48 |  |
| **Self-perceived health** |  |  | 0.679 |  |  |  | 0.016 |  |  |  | 0.320 |  |  |  | 0.001 |
| Very good / Good | 1.00 |  |  |  | 1.00 |  |  |  | 1.00 |  |  |  | 1.00 |  |  |
| Regular | 1.03 | 0.91; 1.18 |  |  | 1.21 | 1.01; 1.45 |  |  | 0.93 | 0.83; 1.04 |  |  | 1.25 | 1.10; 1.41 |  |
| Bad / Very bad | 0.84 | 0.65; 1.08 |  |  | 1.31 | 0.98; 1.75 |  |  | 0.96 | 0.74; 1.24 |  |  | 1.21 | 0.95; 1.54 |  |

Notes: a: excessive alcohol consumption, considering ≥ 5 drinks for males and ≥ 4 drinks for females; b: consumption of processed foods the day before the survey; c: the meeting of physical activity recommendations considers ≥ 150 min per week of moderate physical activity, ≥ 75 min per week of vigorous physical activity, or an equivalent combination of moderate and vigorous physical activity; d: weighted percentage of the sample that spends at least four hours on the investigated outcomes; OR: odds ratio; 95% CI: 95% confidence interval; e: p-value from the Wald heterogeneity test; f: p-value from the Wald test for linear trend; adjusted analysis for sex, age, marital status, skin color, and region of Brazil (first level); education level (second level); smoking, alcohol consumption, processed foods, leisure-time physical activity, and physical activity recommendations (third level); obesity, diabetes, and arterial hypertension (fourth level); self-perceived health (fifth level).
